# Availability of Essential Medicines for Pediatric Oncology in Armenia

**DOI:** 10.31557/APJCP.2019.20.4.991

**Published:** 2019

**Authors:** Tamara Simonyan, Ruzanna Papyan, Samvel Danielyan, Lilit Sargsyan, Varduhi Grigoryan, Hakob Topchyan, Arpina Azaryan, Armen Tananyan, Armen Muradyan, Gevorg Tamamyan

**Affiliations:** 1 *American University of Armenia, School of Public Health,*; 2 *Yerevan State Medical University,*; 3 *Armenian Pediatric Hematology and Oncology Group, *; 4 *Hematology Center, *; 5 *Ministry of Health of the Republic of Armenia, *; 6 *Scientific Center of Drug and Medical Technology Expertise, *; 7 *National Center of Oncology, Yerevan, Armenia. *

**Keywords:** Oncology, cancer, Armenia

## Abstract

**Background:**

One of the main contributors in low survival rate in LMIC is the lack of availability of cancer medications for curative, supportive and palliative care. In many developing countries access to cytotoxic medicine is a major challenge. The information about the availability of essential medicines for pediatric cancer in the country is not known. The main objective of this study was to determine whether the medications used during the treatment of pediatric cancer are available in Armenia.

**Methods:**

In summer 2016 we conducted a survey in the 3 main pharmacies in Yerevan, which import pediatric cancer medications to Armenia to evaluate whether medications used during cancer treatment are officially registered and available in the country. In addition, the information on official registration was cross-checked with the Ministry of Health of the Republic of Armenia (MOH). Simultaneously, detailed information about the drugs, on type of produced drug company, doses and price intervals was confined from the price lists of the national drug importer companies.

**Results:**

The survey included 64 agents in three classes of medication: anti-neoplastics, anti-microbials, and drugs used in supportive care. All of these medications were included in the recent version of the WHO model list of essential medicines. From 30 anti-neoplastic medications on the essential medicines list 22 (73%) were officially registered in Armenia; from 19 anti-microbial drugs all were registered except caspofungin and from 15 supportive care agents 13 (87%) were registered. From registered anti-neoplastic drugs 18% and from antimicrobial drugs 33% were not available in the drug stores.

**Conclusion:**

**T**his study showed that not all the drugs from the SIOP PODC Essential Medication list for pediatric oncology are officially registered and available in Armenia, and effective drug regulation focusing on the childhood cancer care medicine is needed for improving the situation in the country.

## Introduction

Cancer is a one of the leading causes of morbidity and mortality among children until 15 years of age (Ward et al., 2014). Approximately 160,000 new cases of childhood cancer are registered globally each year (Rodriguez-Galindo et al., 2015), and during last 20 years there was around 13% rise in the global incidence of childhood cancer (Steliarova-Foucher et al., 2017). Recent decades’ progress in science and medicine made crucial changes in the outcomes of childhood cancer. Currently, in high-income countries, where around 20% of global pediatric cancer cases are reported, through the implementation of modern diagnostic and treatment methods, policy changes for increasing early detection, the long-term survival of pediatric cancer reaches up to 85% and more (Howard et al., 2008). However, the overall picture is totally different in low- and middle- income countries (LMIC), where the vast majority of pediatric cancer patients reside: in LMIC pediatric cancer survival can range from 0 up to 50-60%. A number of reasons contribute for such inferior outcomes: under and delayed diagnosis, treatment refusals and abandonment, lack of access to surgery, delivering chemotherapy and/or radiotherapy, limitations in supportive care, shortage of specialists, lack of multidisciplinary cancer management teams, and others (Rodriguez-Galindo et al., 2015; Rodriguez-Galindo et al., 2013; Ribeiro et al., 2016; Eden, 2012; Mehta et al., 2013; Denburg et al., 2017; Barr and Robertson, 2016). 

Among them, one of the main contributors in low survival rate in LMIC is the lack of availability of cancer medications for curative, supportive and palliative care. In many developing countries access to cytotoxic medicine is a major challenge. Therefore, achieving equitable access to affordable medication will significantly improve childhood cancer outcomes in developing world (Rodriguez-Galindo et al., 2015; Rodriguez-Galindo et al., 2013; Eden, 2012; Denburg et al., 2017; Barr and Robertson, 2016; Robertson et al., 2015). 

The Pediatric Oncology in Developing Countries (PODC) committee of the International Society of Pediatric Oncology (SIOP), originated a working group to set the list of essential medicine in LMIC’s. The group separated 51 agents in three groups of medication: anti-neoplastics, anti-microbials and supportive care agents based on the 3rd WHO model list of essential medicines for children. During selection of medications the prices of drugs, resource limitations and inequalities in health care system in LMIC’s were considered. In addition, 13 agents were recommended for more advanced care for those countries that are LMIC’s but performed substantial improvement in childhood cancer treatment (Mehta et al., 2013). 

In Armenia childhood cancer statistics reported by the National Institute of Health (no cancer registry exists in the country) are mostly comparable with the world statistics. Our recent small reports showed significant therapeutic and diagnostic limitations, as well as diagnosis delay for pediatric cancer in Armenia (Safaryan et al., 2017). However, the information about the availability of essential medicines for pediatric cancer in the country is not known. The main objective of this study was to determine whether the medications used during the treatment of pediatric cancer are available in Armenia. 

## Materials and Methods

In summer 2016 we conducted a survey in the 3 main pharmacies in Yerevan, which import pediatric cancer medications to Armenia to evaluate whether medications used during cancer treatment are officially registered and available in the country. In addition, the information on official registration was cross-checked with the Ministry of Health of the Republic of Armenia (MOH). Simultaneously, detailed information about the drugs, on type of produced drug company, doses and price intervals was confined from the price lists of the national drug importer companies. The list of essential drugs for pediatric oncology in developing countries, created by the International Society of Pediatric Oncology (SIOP), Pediatric Oncology in Developing Countries (PODC) committee in 2013 was used as a reference (Mehta et al., 2013).

To evaluate the results of the study, descriptive statistical methods were incorporated.

## Results

The survey included 64 agents in three classes of medication: anti-neoplastics, anti-microbials, and drugs used in supportive care. All of these medications were included in the recent version of the WHO model list of essential medicines (Mehta et al., 2013). 

From 30 anti-neoplastic medications on the essential medicines list 22 (73%) were officially registered in Armenia. The non-registered drugs were 13-cis retinoic acid, All-trans retinoic acid, busulfan, daunorubicin, imatinib, melphalan, thioguanine and topotecan. From 19 anti-microbial drugs all were registered except caspofungin (Table 1). 

From 15 supportive care agents 13 (87%) were registered; the non-registered ones were codeine and docusate. IV morphine is registered in Armenia and is provided free by prescription for children with cancer. (Table 1). However, at the time of survey there was a shortage of IV morphine at the hospitals. Oral morphine was not registered in Armenia at the time of our survey.

From registered anti-neoplastic drugs 18% were not available in the drug stores (dacarbazine, irinotecan, methylprednisolone, vinorelabine). From registered anti-microbial drugs 33% (amphotericin B, cefepime, ceftazidime, gancyclovir, vancomycin, voriconazole) and from supportive care drugs only one drug (gabapentine) were not available. 

## Discussion

To the best of our knowledge, this is the first study to evaluate the availability of essential medications for pediatric oncology in Armenia.

**Table 1:** Availability and Registration Status of Medications for the Treatment of Children with Cancer in Low- and Middle-Income Countries in Armenia (Adapted from Mehta et al., 2013)

Anti-neoplastics	Registered	Available	Anti-microbials	Registered	Available	Supportive care agents	Registered	Available
Asparaginase			Acyclovir			Allopurinol		
Bleomycyin			Amikacin			Amitriptyline		
Carboplastin			Amoxicillin			Codeine		
Cisplatin			Amphotericin B			Diazepam		
Cyclophosphamide			Ampicillin			Docusate		
Cytarabine			Cefotaxime			Folinic acid		
Dacarbazine			Ceftazidime			Heparin		
Dactinomycin			Ceftriaxone			Lactulose		
Daunorubicin			Clindamycin			MESNA		
Dexamethasone			Fluconazole			Metoclopramide		
Doxorubicin			Gentamicin			Midazolam		
Etoposide			Meropenem			Morphine[1]		
Hydrocortisone			Metronidazole			Ondansetron		
Hydroxyurea			Trimethoprim – selfamethaxozole			Senna		
Ifosfamide			Vancomycin					
Mercaptopurine								
Methotrexate								
Methylprednisolone								
Prednisolone								
Thioguanine								
Vinblastine								
Vincristine								
Additional drugs for advanced care								
All trans retinoic acid			Caspofungin			Gabapentin		
13 cis retinoic acid			Cefepime					
Busulfan			Gancyclovir					
Imatinib			Voriconazole					
Irinotecan								
Melphalan								
Topotecan								
Vinorelbine								

At the time of survey only IV morphine was registered, and there was a shortage at the hospitals

The study showed that not all the drugs from the SIOP PODC Essential Medication list for pediatric oncology are officially registered and available in Armenia, which proves the luck of access for those medications in the country.

Our research addresses issues of cancer care medicine shortages in Armenia, which as a wide concept and includes unavailability and unaffordability of drugs. Lack of access of medicine because of an expensiveness of drugs is a main representative of unaffordability. 

We have concluded that childhood cancer medicine in Armenia is also affected by shortages, as in many countries in the world. In the report from the European Association of Hospital Pharmacists, based on the results of the survey from 36 countries, 55% of respondents informed shortages of cancer medicines (European Association of Hospital Pharmacists, 2014; The Economist Intelligence Unit, 2017). 

Unavailability and unaffordability of drugs are closely related with non-registration. Registration of drugs ensures safety and quality of medicine, giving opportunity to regulate and monitor the pharmaceutical market and control what it supplies. Following our results, as well as considering the reports from the other countries (Rodriguez-Galindo et al., 2013; Denburg et al., 2017; Barr and Robertson, 2016), we would recommend, that effective drug regulation focusing on the childhood cancer care medicine is needed for improving the situation in the country.

## Statement conflict of interest

The authors has stated that they have no conflicts of interest.

## References

[B1] Barr R, Robertson J (2016). Access to cytotoxic medicines by children with cancer: A focus on low and middle income countries. Pediatr Blood Cancer.

[B2] Denburg A, Arora B, Arora RS (2017). Access to essential medicines for children with cancer: a joint SIOP-CCI position statement. Lancet Oncol.

[B3] Eden Tim (2012). Curing pediatric cancer in the developing world.

[B4] European Association of Hospital Pharmacists (2014). Medicines Shortages in European Hospitals. Brussels.

[B5] Howard SC, Metzger ML, Wiliams JA (2008). Childhood cancer epidemiology in low-income countries. Cancer.

[B6] Mehta PS, Wiernikowski JT, Petrilli JA, Barr RD, Working Group on Essential Medicines of the Pediatric Oncology in Developing Countries committee of SIOP (2013). Essential medicines for pediatric oncology in developing countries. Pediatr Blood Cancer.

[B7] Ribeiro RC, Antillon F, Pedrosa F, Pui CH (2016). Global pediatric oncology: Lessons from partnerships between high-income countries and low- to mid-income countries. J Clin Oncol.

[B8] Robertson J, Magrini N, Barr R, Forte G, Ondari C (2015). Medicines for cancers in children: The WHO model for selection of essential medicines. Pediatr Blood Cancer.

[B9] Rodriguez-Galindo C, Friedrich P, Alcasabas P (2015). Toward the cure of all children with cancer through collaborative efforts: Pediatric oncology as a global challenge. J Clin Oncol.

[B10] Rodriguez-Galindo C, Friedrich P, Morrissey L, Frazier L (2013). Global challenges in pediatric oncology. Curr Opin Pediatr.

[B11] Safaryan L, Sargsyan L, Hakobyan L (2017). Diagnostic and therapeutic limitations and delayed diagnosis of pediatric hematologic malignancies in Armenia: A single-institution report. Clin Lymphoma Myeloma Leuk.

[B12] Steliarova-Foucher E, Colombet M, Ries LAG (2017). International incidence of childhood cancer, 2001-10: a population-based registry study. Lancet Oncol.

[B13] The Economist Intelligence Unit (2017). Cancer medicines shortages. Europe Policy recommendations to prevent and manage shortages.

[B14] Ward E, DeSantis C, Robbins A, Kohler B, Jemal A (2014). Childhood and adolescent cancer statistics 2014. CA Cancer J Clin.

